# A high-dose inoculum size results in persistent viral infection and arthritis in mice infected with chikungunya virus

**DOI:** 10.1371/journal.pntd.0010149

**Published:** 2022-01-31

**Authors:** Yue Zhang, Hu Yan, Xian Li, Dihan Zhou, Maohua Zhong, Jingyi Yang, Bali Zhao, Xuxu Fan, Jun Fan, Jiayi Shu, Mengji Lu, Xia Jin, Ejuan Zhang, Huimin Yan

**Affiliations:** 1 Mucosal Immunity Research Group, State Key Laboratory of Virology, Wuhan Institute of Virology, Chinese Academy of Sciences, Wuhan, China; 2 University of Chinese Academy of Sciences, Beijing, China; 3 Vaccine and Immunology Research Center, Translational Medical Research Institute, Shanghai Public Health Clinical Center, Fudan University, Shanghai, China; 4 Department of Pathology, Union Hospital, Tongji Medical College, Huazhong University of Science and Technology, Wuhan, China; 5 Institute for Virology, University Hospital of Essen, University of Duisburg-Essen, Essen, Germany; 6 Medical Science Research Center, Zhongnan Hospital of Wuhan University, Wuhan, China; Beijing Children’s Hospital Capital Medical University, CHINA

## Abstract

Chikungunya virus (CHIKV) is an emerging mosquito-transmitted alphavirus that leads to acute fever and chronic debilitating polyarthralgia. To date, the mechanism underlying chronic recurrent arthralgia is unknown. In the present study, newborn wild-type C57BL/6 mice were infected with CHIKV, and the virological and pathological features of CHIKV infection were analyzed over a period of 50 days. Acute viral infection was readily established by footpad inoculation of CHIKV at doses ranging from 10 plaque forming unit (PFU) to 10^6^ PFU, during which inoculation dose-dependent viral RNA and skeletal muscle damage were detected in the foot tissues. However, persistent CHIKV was observed only when the mice were infected with a high dose of 10^6^ PFU of CHIKV, in which low copy numbers (10^3^−10^4^) of viral positive strand RNA were continuously detectable in the feet from 29 to 50 dpi, along with a low level and progressive reduction in virus-specific CD8+ T cell responses. In contrast, viral negative strand RNA was detected at 50 dpi but not at 29 dpi and was accompanied by significant local skeletal muscle damage at 50 dpi when mild synovial hyperplasia appeared in the foot joints, although the damage was briefly repaired at 29 dpi. These results demonstrated that a high viral inoculation dose leads to viral persistence and progression to chronic tissue damage after recovery from acute infection. Taken together, these results provide a useful tool for elucidating the pathogenesis of persistent CHIKV infection and viral relapse-associated chronic arthritis.

## Author summary

CHIKV infection has caused several outbreaks and affected millions of people in epidemic areas since the virus was originally isolated in 1952. CHIKV is transmitted by mosquitoes, resulting in acute diseases typically characterized by severe and debilitating arthritis, myalgia and fever, followed by persistent or recurrent joint and muscle pain that may last for months to years. The persistence of CHIKV in patients has been validated by clinical evidence, but it remains unclear how chronic and recurrent tissue injury are correlated with persistent viral infection. In this study, we established acute and chronic CHIKV infections in mice with different viral inoculation doses and identified the correlation of infection dose with outcomes resulting from CHIKV infection. We found that a high inoculation dose not only resulted in persistent viral infection but also led to a typical acute-recovery-relapse model of myositis and joint injury in mice. We also found that CHIKV RNA replication was interrupted after the acute phase, which might be related to the low antiviral T cell responses and relapse of histopathological injuries. Our study highlights the key factors involved in the clinical outcomes after CHIKV infection and provides new insights into the interaction between viral persistence and recurrent arthritis.

## Introduction

Chikungunya virus (CHIKV) is a mosquito-borne virus that causes illness characterized by debilitating arthritis and myositis in humans. CHIKV is a single-stranded positive-sense RNA virus belonging to a group of arthritogenic alphaviruses. CHIKV infections have affected millions of people in epidemic areas, leading to significant public health concerns [1,2]. Over 75% of people bitten by CHIKV-carrying mosquitos suffer from acute disease, typically characterized by severe and debilitating arthralgia/arthritis, myalgia, fever, and sometimes rash [3,4]. While viremia and acute symptoms diminish within a week [5], approximately 30–60% of patients infected with CHIKV manifest persistent or recurrent joint and muscle pain that may last for months to years after the initial diagnosis [6,7]. It is still unclear how CHIKV infection induces persistent/recurrent arthritis despite a robust immune response.

A few studies have reported that CHIKV RNA and proteins can be detected in perivascular synovial macrophages and muscle tissues from patients who have been infected with CHIKV [8,9]. In several CHIKV-infected mouse or macaque models, CHIKV RNA has been persistently detected at low levels in spleen, synovial tissues and foot tissues for 30 days and even up to 150 days [10–12]. One study revealed that CHIKV persisted predominantly in myofibers and dermal and muscle fibroblasts for at least 112 days in infected mice [13]. These studies indicated the persistence of CHIKV in the local muscle or joint tissues, but there is no evidence that the virus actively replicates and produces infectious viral particles during this persistence.

Virus-induced immune responses play a double-edged sword role in viral clearance and immunopathology during the acute stage of CHIKV infection. Immune cells, including intradermal γδ-T cells, macrophages, NK cells, CD4+ and CD8+ T cells, contribute to viral control [12,14,15]. In addition, macrophages and CD4+ T cells participate in CHIKV-induced immunopathology [16,17]. In contrast to CHIKV-induced acute disease, the roles of immune cells in chronic CHIKV pathogenesis are poorly understood. Several studies have indicated that the age of the host and the viral load of the inoculation may be related to viral persistence and the occurrence of chronic arthritis in mice or macaques [10,11,18]. However, it is still unclear how chronic and recurrent tissue injuries are correlated with persistent viral infection.

In the present study, we established a model of immune-competent mice with different viral inoculation doses of a CHIKV isolate derived from a patient in China to investigate how CHIKV induced persistence infection and chronic tissue injury. Using the persistently infected mice, we described the virologic, pathological and immunological kinetics during the whole infection period, and further concentrated on the potential relationship between viral replication and viral-specific immune responses during chronic and recurrent tissue injury. Our results suggest a dynamic interaction between viral replication and pathological outcomes in CHIKV persistently infected mice.

## Materials and methods

### Ethics statement

All animal experiments were conducted in an ABSL-3 facility in biological safety cabinets under a biosafety protocol reviewed and approved by the Ethics and Biosafety Committees, and the Institutional Review Board (IRB), WIV, CAS (permission number: WIVA09201706).

### Mice

Pregnant C57BL/6 wild type (WT) mice were purchased from the Beijing Vital River Laboratory Animal Technology (Beijing, China) at 12 weeks of age. The mice were kept in level-3 isolators (Isocage, Tecniplast, Italy) in an animal biosafety level 3 (ABSL-3) facility at the Wuhan Institute of Virology (WIV), Chinese Academy of Sciences (CAS), according to the Regulations for the Administration of Affairs Concerning Experimental Animals in China (1988) and the Guidelines for Animal Care and Use, WIV, CAS. New-born mice were housed with the mother until they were 21 days old.

### Virus

The CHIKV strain (KC488650) used in the present study was obtained from the National Virus Resource Center, WIV, CAS. This virus, isolated from a clinically CHIKV-positive patient in China, belongs to the Asian lineage and is a sister strain to the strain 0706aTw isolated in Indonesia in 2007, as previously described [19]. The virus was passaged once in BHK-21 cell cultures. CHIKV was amplified and titrated by plaque assay using BHK-21 cells [19]. All infection experiments were performed in a biosafety level-3 (BSL-3) laboratory.

### Mouse infection and sampling

**Fig 1:**
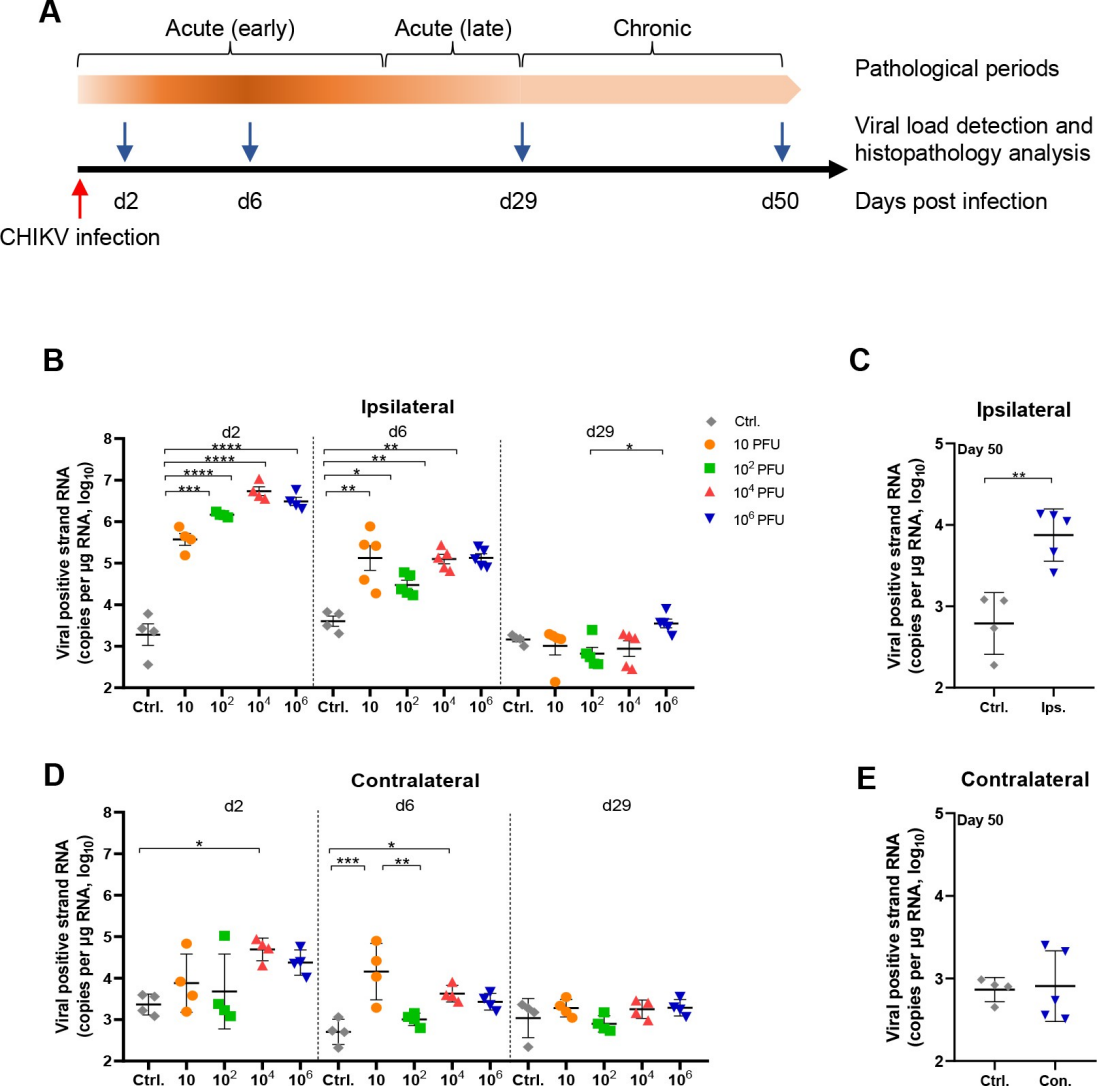
Viral replication in mice infected with different viral doses. Neonate C57BL/6 mice (6–8 days old) were infected by intradermal injection with 10−10^6^ PFU of CHIKV. (A) Schematic diagram of the experimental design and the course of disease. Expression of viral positive strand RNA in the (B) ipsilateral and (D) contralateral feet was detected at 2, 6, and 29 dpi in all groups or (C, E) at 50 dpi in the high dose group. All the samples were assessed by qRT–PCR using nsP1-specific primers and probes. Ctrl, control mice inoculated with BHK-21 cell supernatant. n = 4–5 mice per group. All data are presented as the mean ± SD. *: p< 0.05; **: p< 0.01; ***: p< 0.001; statistical relevance was determined by one-way ANOVA (B, D) or unpaired t test (C, E).

In accordance with the dose range that resulted in different outcomes after infection of the nonhuman primate model [10], suckling mice (6–8 days old) were intradermally inoculated with 10 μl of PBS diluted CHIKV (containing 10 PFU, 10^2^ PFU, 10^4^ PFU, or 10^6^ PFU in 10 μl PBS) into the footpad of the left hindlimb. Control mice were inoculated with PBS-diluted BHK-21 cell supernatant via the same route. According to the viral load and histopathological changes in previously reported mouse models, the disease process is artificially divided into three stages: the early acute stage (0–14 days, high level of viremia and strong tissue damage), late acute stage (15–30 days, decreased viral load and reduced tissue damage), and chronic stage (>30 days) (Fig 1A). At 2, 6, 29 and 50 days postinfection (dpi), 4–6 mice in each group were sacrificed to monitor the virological and pathological changes in each stage. Samples of both sides of the hindlimb feet and knees were obtained for further analysis. For the reinfection experiment, CHIKV-infected mice were inoculated again with 10^4^ PFU viruses in the footpad of the contralateral (right) side. Control mice in the first round of infection were also inoculated with 10^4^ PFU viruses at the contralateral side as controls for reinfection.

For histologic examinations, the feet or knees from 5 mice in each group were fixed in 10% neutral buffered formalin. For RNA extraction and viral plaque assay, tissue samples were weighed and homogenized twice at 5000 rpm for 20 s by a Tissue Cell destroyer D1000 (NZK Ltd., Wuhan, China) in 500 μl diluent (DMEM supplemented with 2% FBS) per 100 mg tissue. The homogenates were stored at -80°C.

### Plaque assay

Samples of viral stock and tissue homogenates were titrated by plaque assay in monolayer cultures of BHK-21 cells as described previously [19,20]. Serial 10-fold dilutions of viral stock or 4-fold dilutions of tissue homogenate supernatant were prepared, and 200 μl of each dilution was added to individual wells. Duplicate wells of BHK-21 cells were inoculated with each dilution. After virus absorption for 1 hour, a layer of 1.25% methyl cellulose (Sigma–Aldrich, USA) was added. Cells were incubated for 48 hours at 37°C with 5% CO_2_, fixed with 4% paraformaldehyde-PBS for 30 min, and stained with 1% crystal violet for 4 h. Plaque morphology and numbers were recorded after washing the plates with tap water.

### Plaque reduction neutralization test (PRNT)

Serum from control or CHIKV-inoculated mice was inactivated at 56°C for 30 min, and 200 μl serum was diluted with DMEM in a 2-fold serial dilution. CHIKV was adjusted to 100 PFU in 200 μl and then added to the diluted serum to mix and incubate at 37°C for 1 h. The virus-antibody mixture was added to appropriate wells of BHK-21 cells and incubated at 37°C for 1.5 h. Cells were then rinsed twice, and a layer of 1.25% methyl cellulose was added. The cells were further incubated and fixed, and plaque numbers were recorded as described in the plaque assay. CHIKV incubated with DMEM was used as a negative control. The percentage of inhibition was calculated as follows: %Inhibition = (number of plaques from the negative control—number of plaques from the serum samples)/number of plaques from the negative control.

### Histopathology examinations

Foot or knee samples were fixed in 10% neutral buffered formalin for at least 48 h. After fixation, the tissue samples were decalcified with EDTA-containing solution for 15 days and then transferred to 70% ethanol, after which they were processed by routine paraffin embedding. Two 5-μm longitudinal sections were stained using hematoxylin and eosin (H&E) and immunohistochemistry (IHC). For IHC staining of E2 protein, feet or knee sections were stained with an anti-CHIKV E2 polyclonal antibody (pAb, 1:100, IBT Bioservices, Rockville, US) or an isotype control antibody, followed by a horseradish peroxidase (HRP)-conjugated goat anti-rabbit IgG (1,200, Invitrogen, USA), and visualized by the Envision System (Dako, Denmark) [10].

### Quantitative RT–PCR detection of viral RNA

Total RNA was isolated from 50–100 μl of tissue homogenate using an RNA isolator reagent (Vazyme Biotech, Nanjing, China). cDNA was prepared from the total RNA with a HiScript II 1st Strand cDNA Synthesis Kit (Vazyme Biotech, Nanjing, China).

To detect the viral positive or negative strand RNA, first strand cDNA was synthesized using the corresponding primers [13,21]. Real-time PCR was performed with AceQ Universal U+ Probe Master Mix (Vazyme Biotech, Nanjing, China). All primers and probes used for RT–PCR are listed in S1 Table. The cycling conditions were 37°C (2 min) for degradation of the remaining RNA, 95°C (5 min) for initial activation, followed by 45 cycles of 95°C (15 s) for denaturation and 60°C (30 s) for annealing and extension. Standard curves were constructed with threshold cycle (Ct) values obtained using the plasmids containing nsP1 and E1. The expression of CHIKV RNA in tissues was calculated using standard curves and normalized to the amount of input RNA. The cutoff value was determined by the results of control mice.

### Detection of serum anti-E2 antibody by enzyme-linked immunosorbent assay (ELISA)

Truncated CHIKV E2 protein (1–350 aa) was prepared by prokaryotic expression in *E*. *coli* strain BL21 (DE plus) as described previously [22]. Each well of flat-bottom 96-well ELISA plates (Greiner, Germany) was coated with truncated E2 protein (5 μg/ml) in carbonate-bicarbonate buffer at 4°C overnight. After blocking with 10% FBS, serial 10-fold diluted samples were added and incubated at 37°C for 2 hours. HRP-labeled goat anti mouse IgG (H+L) (1:5000, Invitrogen, USA) was incubated at 37°C for 1 hour followed by TMB substrate and stop solution for color development (Invitrogen, USA). The absorbance was read at 450 nm with a microplate reader (Thermo Labsystems, USA).

### Analysis of virus-specific T cell function by flow cytometry

Splenocyte suspensions were prepared by homogenization as described previously [23]. For stimulation of splenocytes, cells were stimulated with CHIKV-derived peptides (2 μg/ml), including E1 (HSMTNAVTI), E2 (IILYYYELY) and capsid (ACLVGDKVM), for 4.5 h, as described previously [24]. Cell surface staining was performed using eBioscience or Biolegend reagents, including anti-CD4 (L3T4) and anti-CD8 (53–6.7) antibodies. Dead cells were excluded by Fixable Viability Dye (FVD, eBioscience, USA) staining. For intracellular staining, cells were permeabilized using the Cytofix/Cytoperm intracellular staining kit (eBioscience, USA) and stained with anti-IFN-γ antibody (XMG1.2) and anti-TNF-α antibodies (MP6-XT22). A cytomegalovirus (CMV)-derived peptide (YILEETSVM) was used as the control. Stained cells were measured on an LSRFortessa (BD Bioscience, USA), and the data were analyzed using FlowJo software (Tree Star, Ashland, OR). The frequency of CHIKV-specific CD8+ T cells was calculated by subtracting the background value using the CMV peptide control.

### Statistical analysis

Statistical analysis was performed using GraphPad Prism software. Statistical differences were analyzed by unpaired Student t-test, one-way ANOVA or two-way ANOVA tests. The p-values < 0.05 were considered significant (* indicated p< 0.05, ** indicated p< 0.01, *** indicated p< 0.001). All data points are representative of at least three independent experiments.

The details of the materials and methods for cloning and sequencing of the CHIKV fragments, western blotting, and amplification of virus from tissue samples are provided in the supplementary files (S1 Appendix, materials and methods section).

## Results

### A broad range of CHIKV doses can establish viral infection in mice

Previous studies reported that experimental infection in neonate mice (<10 days old) with CHIKV resulted in severe symptoms or even animal death [18]. In contrast to the reported virus strains, transient and slight swelling was occasionally observed in the inoculated foot in some mice 24 h after infection with the KC488650 strain in the present study. Neither significant loss of body weight nor decreases in motor activity were observed in the inoculated mice during the observation period compared with the control mice.

To monitor viral replication, the viral positive strand RNA content, considered as the viral genome equivalent, was analyzed by qRT–PCR. In the inoculated feet (ipsilateral feet) of each group of mice that received different doses of viruses, the viral positive strand RNA reached the highest levels at 2 dpi and dropped to approximately 1×10^5^ copies (per μg RNA) at 6 dpi (Fig 1B, d2 and d6). Then, at 29 dpi, the viral positive strand RNA copy numbers decreased to an undetectable level in all mice except in the mice that received 10^6^ PFU of the virus, in which the viral RNA was detected at a low level of approximately 10^4^ copies (per μg RNA). In the 10^6^ PFU group, the positive strand RNA was maintained at 10^4^ copies (per μg RNA) in the ipsilateral feet until the endpoint of the experiment (Fig 1C, d50). By cloning CHIKV RNA from the 50 dpi samples, several fragments, including the CDS region of nsP1 and partial regions of the nsP2 protein and E2 protein, were obtained, further proving the presence of viral RNA during the chronic phase (S1 Appendix, S2 Table, and S1 Fig).

In the noninoculated feet (contralateral feet), the viral positive strand RNA also reached the highest levels at 2 dpi in all groups and then dropped to as low as 4×10^4^ copies (per μg RNA) at 6 dpi (Fig 1D). Unlike in the ipsilateral feet, viral positive strand RNA could not be detected in the contralateral foot tissues at 29 dpi or 50 dpi (Fig 1D and 1E).

### CHIKV infection results in acute or chronic pathological injuries in a dose-dependent manner

**Fig 2:**
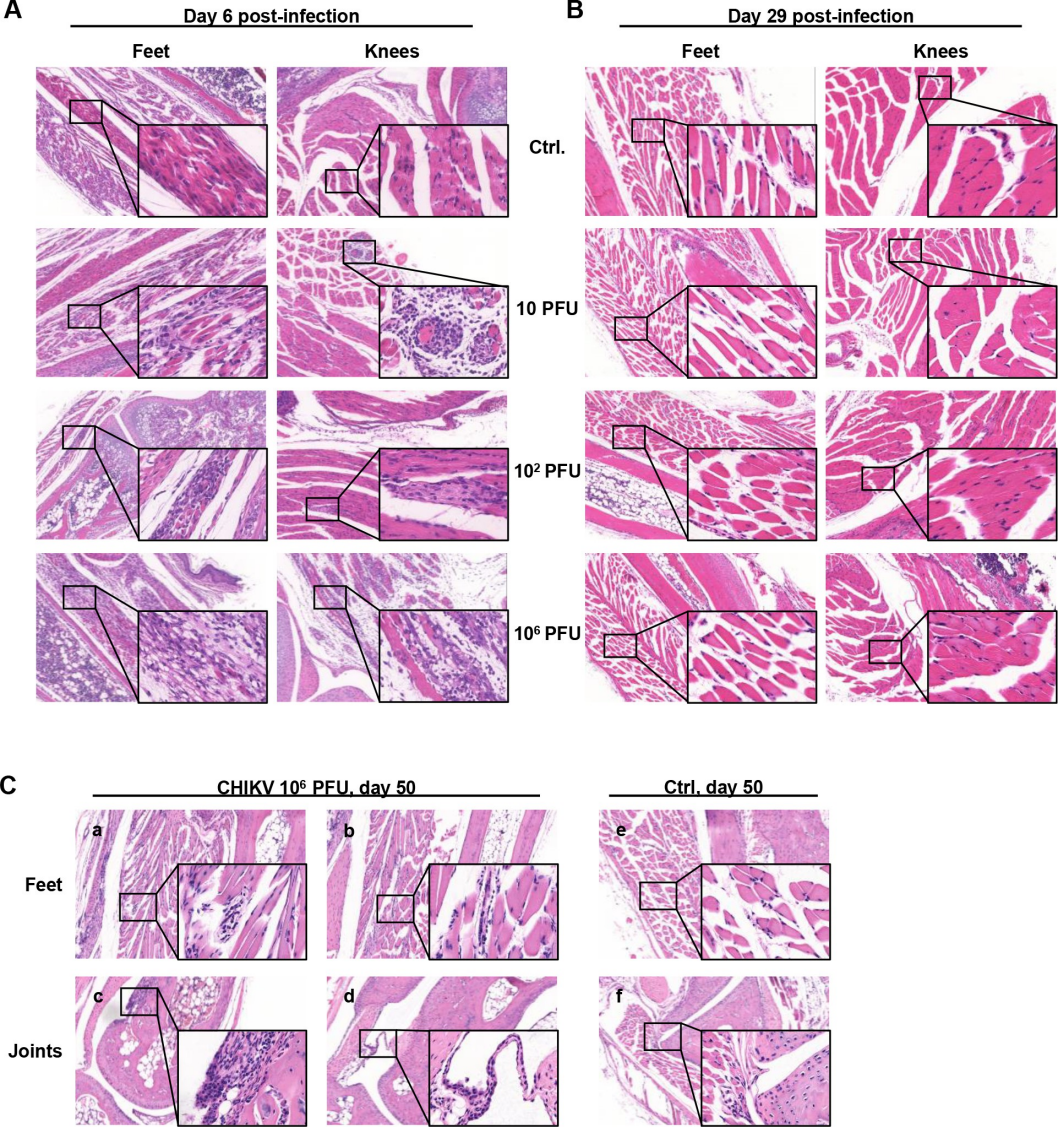
Pathological changes in the feet during acute infection. Neonate C57BL/6 mice (6–8 days old) were infected by intradermal injection with a low dose (10 PFU, 10^2^ PFU) or a high dose (10^6^ PFU) of CHIKV. H&E staining of paraffin-embedded sections of ipsilateral feet and knees was examined at (A) 6 dpi and (B) 29 dpi in all groups and at (C) 50 dpi in the high-dose group. Ctrl, control mice inoculated with BHK-21 cell supernatant. Original images are shown using low magnification (×50) and high magnification (×200). Data are representative of at least 5 mice.

During acute infection, the most significant pathological changes were located in the skeletal muscles of the feet and knees at 6 dpi, marked by degeneration and necrosis of the skeletal muscle and infiltration of mononuclear cells (Fig 2A). In the feet and knees, inoculation with low doses of viruses (10 and 10^2^ PFU) resulted in several small focal areas of muscle cell necrosis surrounded by inflammatory cells. Inoculation with 10^6^ PFU resulted in massive necrosis of muscle cells filled with infiltrated inflammatory cells, leading to the fragmentation of muscle fibers and enlarged spaces between the muscle bundles. Unlike in the muscle tissue, the pathological changes rarely involved the joints.

Neither the feet nor the knee muscles and joints showed obvious pathological changes between the CHIKV-infected mice and the control mice at 29 dpi, indicating that the tissue damage was almost entirely healed in all the mice after the acute phase (Fig 2B). Considering the persistent presence of virus in the tissue, we focused on the high-dose group of mice during the chronic phase. Interestingly, new pathological changes were observed again at 50 dpi in the ipsilateral feet of 10^6^ PFU group mice. These new pathological changes consisted of the degeneration and necrosis of skeletal muscles, which had previously been observed at 6 dpi, and hyperplasia of the joint synovia that was not observed at any earlier time points (Fig 2C). Compared to severe skeletal myositis at 6 dpi, the lesions of the skeletal muscles were mainly small focal or punctate necrosis characterized by muscle cell nuclei arranged as a string of beads with (Fig 2C, panel a) or without (Fig 2C, panel b) a few infiltrated lymphocytes. Synovial hyperplasia appeared in a few of the animals’ feet joints at 50 dpi (Fig 2C, panel c and d).

No obvious lesions were found in the contralateral feet of 10 PFU-inoculated mice, while small foci were found in the contralateral feet of 10^2^ and 10^6^ PFU-inoculated mice only at 6 dpi (S2 Fig). These results indicate that CHIKV infection-induced acute tissue injury on both sides was almost fully repaired within one month. However, inoculation with a high dose of 10^6^ PFU of CHIKV resulted in recurrent and progressive chronic tissue damage in the inoculated site after recovery from acute injury.

### CHIKV RNA replication is intermittent and active at low levels during the chronic injury period

To investigate the reasons for incomplete viral control, we tried to isolate the virus from chronically infected tissues. Infectious viral particles could not be directly observed by plaque assay. Amplification of virus from tissue homogenate with BHK-21 cells failed, with no infectious CHIKV particles and no detectable CHIKV RNA after serial passages for 5–8 generations. Moreover, CHIKV proteins were undetectable by western blotting or IHC staining at 29 and 50 dpi. These results suggest a very low rate of viral replication in the tissue after the acute phase.

**Fig 3:**
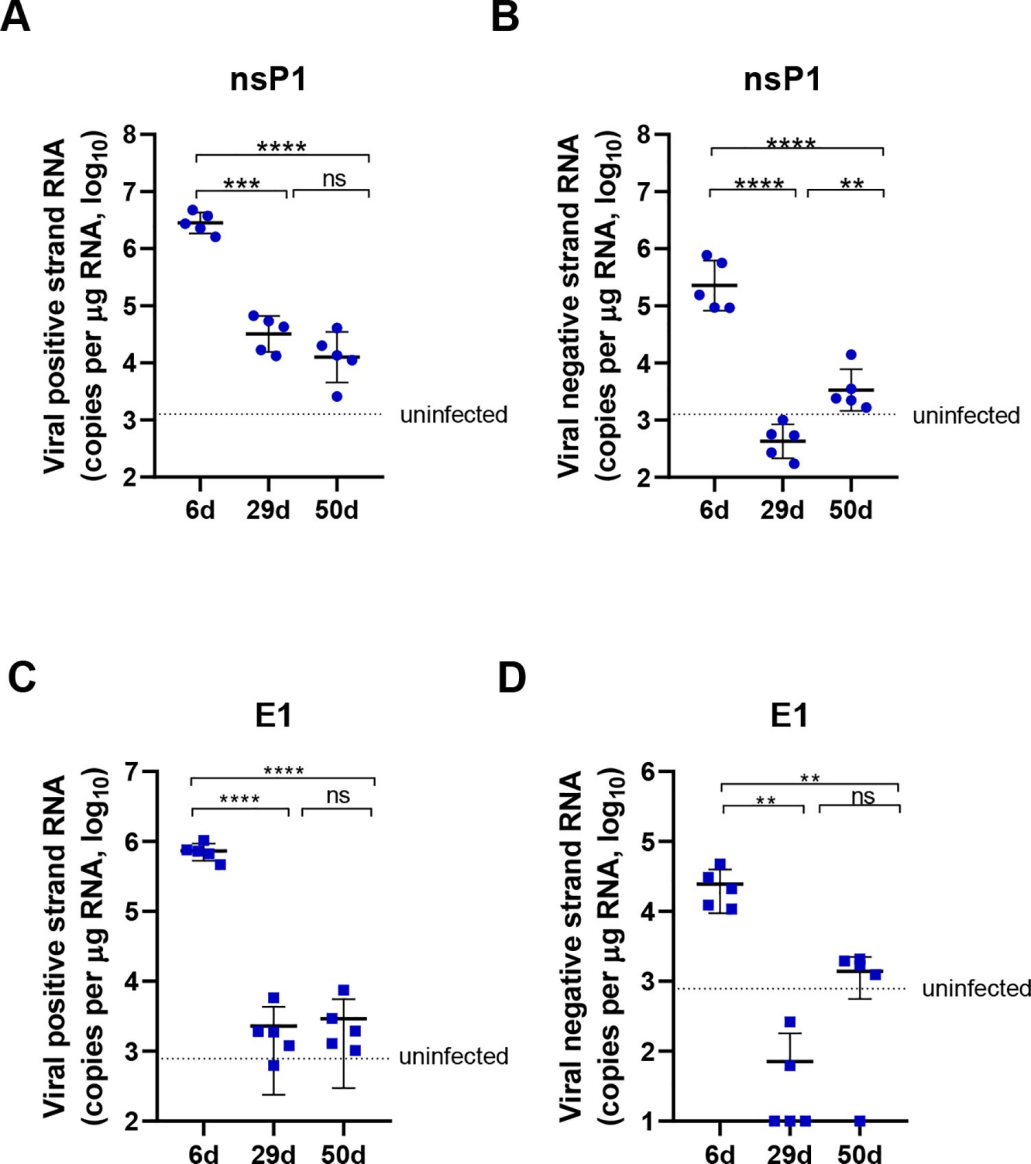
Intermittent viral replication in the foot tissue. Neonate C57BL/6 mice (6–8 days old) were infected by intradermal injection with 10^6^ PFU of CHIKV. The mice were sacrificed at 6, 29 or 50 dpi. (A, B) Expression of the positive strand and negative strand RNA in the ipsilateral feet was assessed by qRT–PCR using primers and probes specific for the nsP1 gene. (C, D) Expression of the positive strand and negative strand RNA in the ipsilateral feet was detected by qRT–PCR using primers and probes specific for the E1 gene. Dashed line, average value of control mice inoculated with BHK-21 cell supernatant. n = 5 mice per group. All data are presented as the mean ± SD. *: p< 0.05; **: p< 0.01; ***: p< 0.001; statistical relevance was determined by one-way ANOVA.

Then, we determined whether the virus was replicating during the chronic phase. As a positive strand RNA virus, the viral RNA in the infected cells contains the genomic RNA (gRNA, positive strand) and the replication intermediates (double strand). Therefore, the positive and negative strand RNAs of CHIKV in the nsP1 and E1 regions were analyzed. In the samples derived from the acute phase (6 dpi), a high level of positive strand RNA and a relatively lower level of negative strand RNA were detected in the inoculated feet (Fig 3). Viral positive strand RNA was present at a low level at 29 and 50 dpi in all animals (Fig 3A and 3C). However, the negative strand RNA decreased to an undetectable level at 29 dpi but reoccurred at 50 dpi at a low level in all mice (Fig 3B and 3D). The fluctuating level of negative strand RNA implies that CHIKV replication decreased at 29 dpi and was reactivated at 50 dpi, which indicates an intermittent manner to maintain the persistence of viral genomes even after recovery from acute infection.

### A high dose of CHIKV induces neutralizing antibodies but weak CD8+ T cell responses

**Fig 4:**
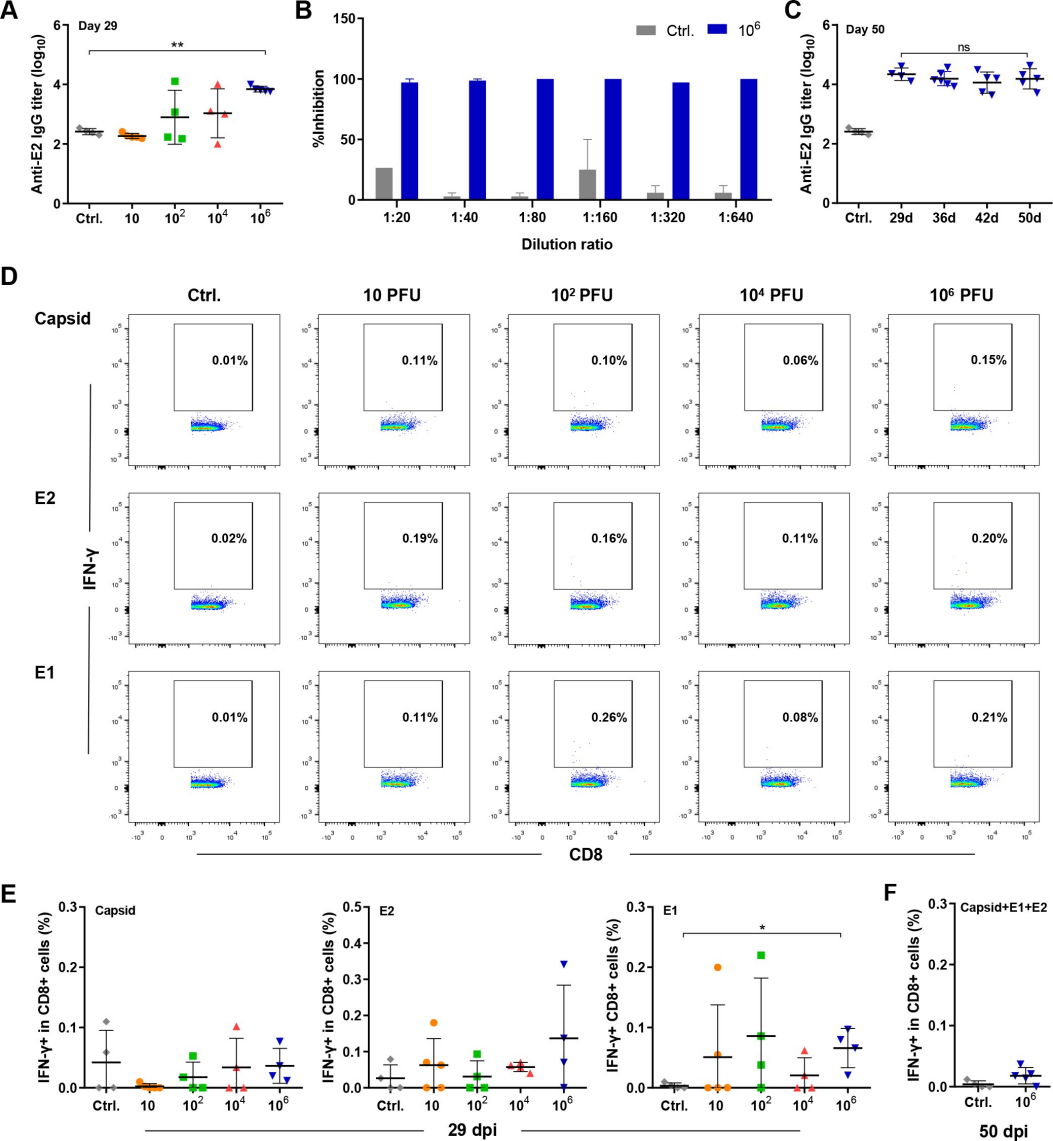
Immune responses induced by CHIKV infection. Neonate C57BL/6 mice (6–8 days old) were infected by intradermal injection with 10−10^6^ PFU of CHIKV. (A) Serum anti-E2 IgG was detected by ELISA in all groups at 29 dpi. (B) The neutralizing capacity of the serum from the mice in the control group and the 10^6^ PFU group was tested by the PRNT assay. (C) Serum anti-E2 IgG in the 10^6^ PFU group was assessed by ELISA at 29, 36, 42 and 50 dpi. (D, E) Splenocytes were isolated at 29 dpi or (F) 50 dpi and stimulated with CHIKV-derived capsid, E2 and E1 peptides for 4.5 h, and the frequencies of IFN-γ-producing CD8+ T cells were assessed by FACS. Ctrl, control mice inoculated with BHK-21 cell supernatant. n = 4 mice per group. All data are presented as the mean ± SD. *: p< 0.05; **: p< 0.01; ***: p< 0.001; statistical relevance was determined by one-way ANOVA.

Virus-induced adaptive immune responses play an important role in viral clearance and in the protection of animals from reinfection [25,26]. Therefore, we wondered whether viral persistence was related to the deficiency of virus-induced immune responses. All mice infected with 10^6^ PFU CHIKV produced anti-E2 IgG antibodies (Fig 4A), which have the capacity to neutralize CHIKV at a titer of 1:640 (Fig 4B). This anti-E2 antibody titer was maintained at a similar level from 29 dpi until 50 dpi (Fig 4C). No escape mutations were found in the antibody-binding domains of the E2 protein, suggesting that the neutralizing activities of anti-E2 antibodies were not reduced during the chronic phase (S1 Appendix, S2 Table, and S1 Fig). Splenic capsid-, E1- and E2-specific IFN-γ-producing CD8+ T cells were found at a low level in some mice in the low-dose (10−10^4^ PFU) groups and in all of the mice in the high-dose (10^6^ PFU) group at 29 dpi (Fig 4D and 4E). However, these IFN-γ-producing virus-specific CD8+ T cells decreased to a negligible level at 50 dpi (< 0.05% of CD8+ T cells) (Fig 4F). These results suggested that CHIKV infection successfully activated the humoral immune response together with weak and short-lived virus-specific CD8+ T cell responses.

### CHIKV-induced immune responses are not sufficient to eliminate viruses

**Fig 5:**
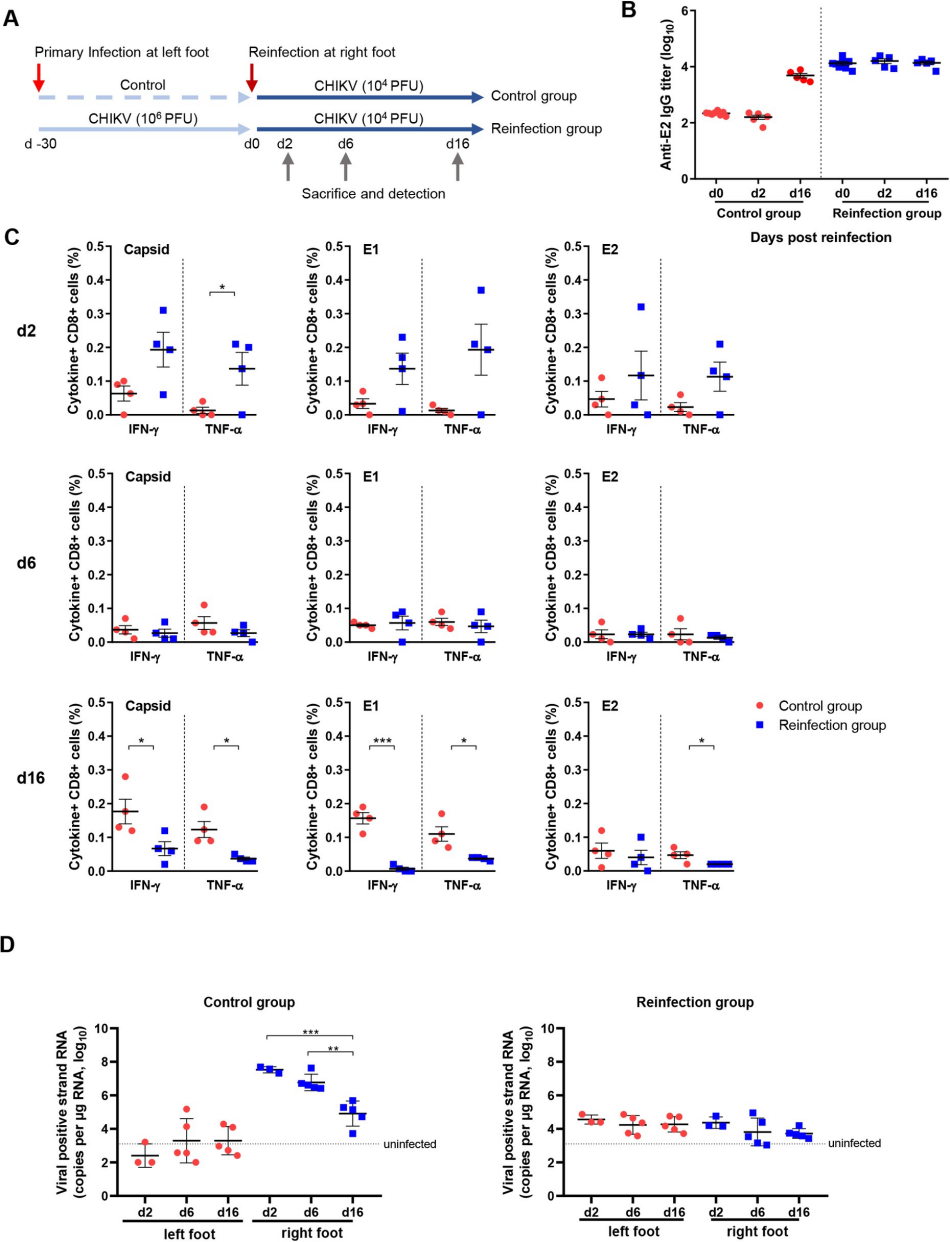
Viral load and immune responses after reinfection. (A) Experimental design of the primary infection and reinfection experiment. (B) Serum anti-E2 IgG was assessed by ELISA at 0, 6, and 16 days after reinfection. (C) Splenocytes were isolated at 2, 6, and 16 days after reinfection and stimulated with CHIKV-derived capsid, E2 and E1 peptides for 4.5 h, and the frequencies of IFN-γ- and TNF-α-producing CD8+ T cells were assessed by FACS. (D) Expression of viral positive strand RNA in feet was assessed by qRT–PCR using nsP1-specific primers and probes. Dashed lines in D, average value of PBS-inoculated mice. n = 4–5 per group. All data are presented as the mean ± SD. *: p< 0.05; **: p< 0.01; ***: p< 0.001; statistical relevance was determined by one-way ANOVA.

To investigate whether virus-induced immune responses have the potential to control viral replication, mice in the high-dose group were reinfected at 30 days after primary infection (Fig 5A). Anti-E2 antibodies remained at a similar level before and after reinfection (Fig 5B). Splenic capsid-, E1- and E2-specific CD8+ T cells were rapidly reactivated and produced antiviral cytokines, including IFN-γ and TNF-α, in most of the reinfected mice at 2 dpi. However, the frequencies of these antiviral T cells rapidly decreased to baseline within 6 dpi and lasted until 16 dpi, indicating the loss of quantity or function of virus-specific CD8+ T cells (Fig 5C). The viral RNA in the control group showed similar kinetics and viral load as the neonate mice during primary infection (Fig 5D, left panel). In contrast, in the reinfected mice, the viral RNA level in the right feet (reinfected side) was below 10^5^ copies per μg RNA, suggesting that the pre-existing immune responses suppressed viral infection and replication in the new infection site. However, viral RNA was present at a similar level in both the right feet (reinfected side) and the left feet (primary infected side) in the reinfected mice (Fig 5D, right panel), suggesting that CHIKV infected and replicated at the new site. These results indicate that CHIKV-induced immune responses reduced viral replication but could not completely clear the viruses or prevent reinfection.

## Discussion

In the present study, we described the kinetics of virological and pathological changes in acute and persistent CHIKV infection as well as CHIKV-induced chronic arthritis in an immune-competent wild-type mouse model. As a mosquito-borne virus, the amount of virus carried by mosquitoes is directly related to the amount of virus that enters the host during the bite. *Ledermann JP*. *et al* reported that CHIKV titers in the saliva of virally infected mosquitoes were variable, ranging from 5×10^2^ to 4×10^4^ PFU [27]. By experimental infection of animals, the results of *Labadie K*. *et al* [10] and our results reveal different sequalae of viral replication and pathological changes that are related to the inoculation doses. We found that exposure to a very low dose of CHIKV was sufficient to establish an acute infection that was resolved within 4 weeks, but only exposure to a high viral inoculum inevitably caused viral persistence and chronic injury that lasted for over 50 days in the local tissue. These studies suggest the potential relationship between CHIKV infection dose, viral persistence and recurrent arthritis symptoms and recapitulate the various clinical symptoms and outcomes of CHIKV infection in humans, which supports the hypothesis that the initial viral inoculum is a key determinant of pathogenesis.

CHIKV infection induces immune activation and the development of protective antiviral immune responses [28–30]. As a result, infection of adult mice with CHIKV leads to acute injury, despite persistent viral RNA in the tissue being described by several reports [12,13]. A chronic tissue injury model was established in neonatal mice but rarely in adults, probably due to the immature immune system in neonates [31]. Our study found that the high-dose (10^6^ PFU) inoculum successfully and rapidly induced an anti-E2 antibody response and virus-specific CD8+ T cell responses, which may contribute to viral control during the acute phase. However, these immune responses provided only partial protection when re-encountering the virus. The low level and short-lived virus-specific CD8+ T cell response might lead to incomplete viral clearance during chronic infection or reinfection. This reduction in antiviral CD8+ T cells may be attributed to the silencing of viral replication or the absence of viral antigens during the chronic phase, as has been reported previously in clinical biopsy and animal models [8].

Viral negative strand RNA is related to active viral replication in both CHIKV-infected mice and cultured cells [32–35]. In the animals of our study, we note that the negative strand viral RNA decreased to a negligible low level and thereafter increased again, which suggests the functionality of the virus replication complex during the chronic phase. Rebound of the negative strand RNA suggested that the viral genome is undergoing low-level active replication in an intermittent manner rather than a persistent manner during the chronic phase. The underlying virological and pathogenic mechanisms of reactivated viral replication during the chronic phase warrant further in-depth investigation.

In contrast to previous CHIKV-infected neonatal mouse models, our results showed a short recovery period of tissue pathology after the early stage of acute infection and recurrent progressing arthritis. The mechanisms of the fluctuating pathological changes are not clear and are presumably related to the weak pathogenicity of this viral strain. Interestingly, CHIKV-negative strand RNA was undetectable during the tissue recovery period but reappeared during the chronic pathological period. The coincident dynamics of the negative strand RNA and the pathological changes suggest that the cessation and reactivation of viral replication might be associated with the recovery and reemergence of pathological symptoms. We speculate that some residual viruses in the muscle tissue allow for the capacity for virus reactivation and induce a second round of tissue damage after the decrease in antiviral immune responses. The underlying specific mechanisms require further studies.

In this study, we reported a mouse model with clear and typical histopathology of disease progression after CHIKV infection, including the acute myositis period, recovery period, and chronic relapse myositis and arthritis period. This model, established based on the neonate mice, could not accurately explain the mechanism of chronicity in the adults. However, the present study provides a useful *in vivo* model for elucidating the pathogenesis of persistent CHIKV infection and viral relapse-associated chronic arthritis. Our findings with this model will also help to further investigate intrinsic changes induced by viral replication in persistent viral reservoir cells.

## Supporting information

S1 Appendix Supporting information of materials and methods section, and results section. (DOCX)

S1 Fig Persistence of viral RNA in the foot tissue. Neonate C57BL/6 mice (6–8 days old) were infected by intradermal injection with 10^6^ PFU of CHIKV or PBS. The mice were sacrificed at 50 dpi. (A) Schematic diagram of the CHIKV fragment amplification region. (B) Three fragments of CHIKV genes were amplified from two RNA samples of ipsilateral feet by nested RT–PCR and verified by agarose gel electrophoresis. (C) Alignment of the nucleotide sequences of the CHIKV gene fragments amplified from RNA samples of the viral stock or ipsilateral feet (50 dpi). The online sequence of CHIKV (GenBank accession No: KC488650) was used as a reference. n = 2 per group.
(DOCX)

S2 Fig Pathological changes in the contralateral feet during the acute stage. Neonate C57BL/6 mice (6–8 days old) were infected by intradermal injection with 10 PFU, 10^2^ PFU, and 10^6^ PFU of CHIKV. (A) H&E staining of paraffin-embedded sections of contralateral feet was examined at 6 dpi. (B) H&E staining of paraffin-embedded sections of contralateral feet was also examined at 29 dpi. Original images are shown at low magnification (×50) and high magnification (×200). n = 5 per group.
(DOCX)

S1 Table Primers and probes of nsP1 and E1 for qRT-PCR detection. (DOCX)

S2 Table Primers for nested-RT-PCR amplification. (DOCX)
