# Supplementary material for: A high-dose inoculum size results in persistent viral infection and arthritis in mice infected with chikungunya virus
Source: PLoS Negl Trop Dis. 2022 Jan 31;16(1):e0010149. doi: 10.1371/journal.pntd.0010149 (PMC8803182; doi:10.1371/journal.pntd.0010149)
Supplement: S1 Appendix — (DOCX) [file pntd.0010149.s001.docx]

**S1 Appendix**

**Materials and methods**

**Cloning of CHIKV RNA fragments**

Total RNA of the ipsilateral foot samples derived from two of the CHIKV-infected mice at 50 dpi was used for cDNA synthesis. The RNA of the viral stock was extracted and used as the control. cDNA was prepared from total RNA using corresponding reverse-transcription primers for each fragment. Fragments were amplified by nested RT–PCR with Super Fidelity DNA Polymerase (Vazyme Biotech, China), cloned into pMD18-T vector (Takara, China) and sequenced. Alignment of the sequences was performed with DNAMAN 8.0 Demo (Lynnon Biosoft). All primers used for PCR are listed in S2 Table.

**Western blotting**

Foot tissue homogenates were lysed using lysis buffer containing 1% SDS and protease inhibitor cocktails. Equal amounts of protein (100 μg) were separated by SDS–PAGE and transferred to polyvinylidene difluoride membranes (Millipore, Germany). After blocking the nonspecific sites with 5% BSA, anti-CHIKV E2 pAb (IBD Bioservices, Rockville, US) was diluted 2000-fold and incubated with the membranes, followed by incubation with HRP-conjugated goat anti-rabbit IgG (1:8000, Jackson ImmunoResearch, USA). Immunoreactive bands were then developed with an enhanced chemiluminescence system (GE Healthcare, Germany).

**Amplification of virus from tissue samples**

The homogenized foot tissues were centrifuged at 4000 rpm for 10 min at 4 °C. BHK-21 cells were infected with the supernatant and cultured at 37 °C and 5% CO_2_ for 48 h. Infected cells were passaged for 8 generations. The cytopathic effect was monitored with a microscope, and one-third of the cells from each generation were harvested for the detection of E2 protein expression by western blotting and viral RNA by qPCR detection.

**Results**

**Sequencing of CHIKV fragments from the chronic phase**

Three fragments were obtained from the inoculated feet at 50 dpi, including fragment 1 (nt 9-863, 854 bp), fragment 2 (nt 806-2149, 1343 bp) and fragment 3 (nt 9380-9614, 234 bp) (S1A and S1B Fig). These fragments covered the complete CDS of nsP1, part of the nsP2 protein, and two monoclonal antibody-binding domains of E2 protein [1], indicating that the viral RNA may be present in the feet in long fragments that likely cover the full length of the viral genome. The nucleotide sequences of the fragments amplified from the infected mice were identical to those amplified from the viral stock in parallel, despite several point mutations in comparison to the reference sequence of CHIKV isolate KC488650 (S1C Fig). These results suggested that immune selection-related nucleotide mutations did not occur during persistent infection.

**Reference**

1. Jin J, Simmons G. Antiviral Functions of Monoclonal Antibodies against Chikungunya Virus. Viruses. 2019;11(4):305. doi:10.3390/v11040305
