# Supplementary material for: A high-dose inoculum size results in persistent viral infection and arthritis in mice infected with chikungunya virus
Source: PLoS Negl Trop Dis. 2022 Jan 31;16(1):e0010149. doi: 10.1371/journal.pntd.0010149 (PMC8803182; doi:10.1371/journal.pntd.0010149)
Supplement: S1 Fig — Neonate C57BL/6 mice (6–8 days old) were infected by intradermal injection with 106 PFU of CHIKV or PBS. The mice were sacrificed at 50 dpi. (A) Schematic diagram of the CHIKV fragment amplification region. (B) Three fragments of CHIKV genes were amplified from two RNA samples of ipsilateral feet by nested RT–PCR and verified by agarose gel electrophoresis. (C) Alignment of the nucleotide sequences of the CHIKV gene fragments amplified from RNA samples of the viral stock or ipsilateral feet (50 dpi). The online sequence of CHIKV (GenBank accession No: KC488650) was used as a reference. n = 2 per group. (DOCX) [file pntd.0010149.s002.docx]

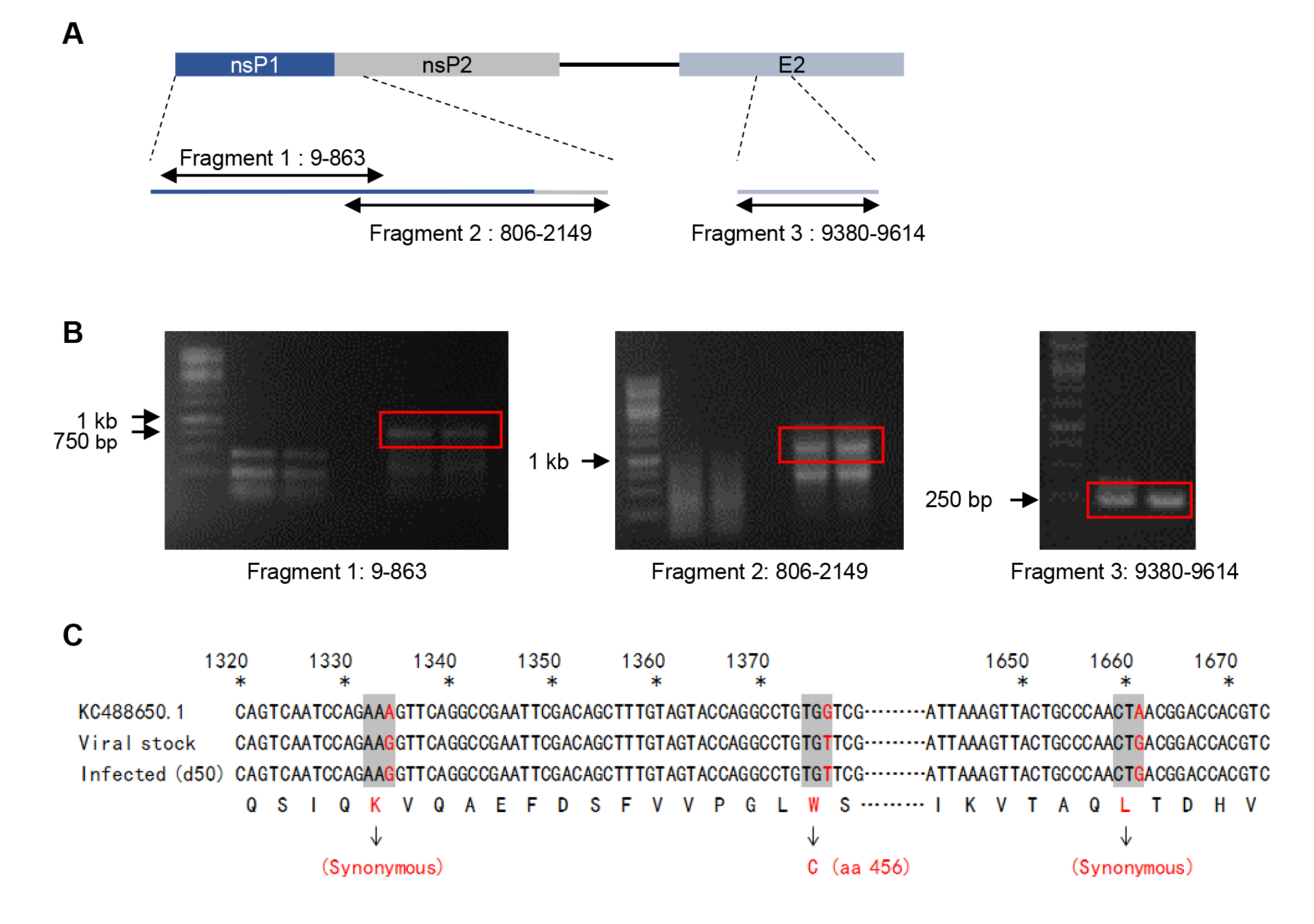


**S1 Fig. Persistence of viral RNA in the foot tissue.** Neonate C57BL/6 mice (6-8 days old) were infected by intradermal injection with 10^6^ PFU of CHIKV or PBS. The mice were sacrificed at 50 dpi. (A) Schematic diagram of the CHIKV fragment amplification region. (B) Three fragments of CHIKV genes were amplified from two RNA samples of ipsilateral feet by nested RT–PCR and verified by agarose gel electrophoresis. (C) Alignment of the nucleotide sequences of the CHIKV gene fragments amplified from RNA samples of the viral stock or ipsilateral feet (50 dpi). The online sequence of CHIKV (GenBank accession No: KC488650) was used as a reference. n=2 per group.
