# Supplementary material for: A high-dose inoculum size results in persistent viral infection and arthritis in mice infected with chikungunya virus
Source: PLoS Negl Trop Dis. 2022 Jan 31;16(1):e0010149. doi: 10.1371/journal.pntd.0010149 (PMC8803182; doi:10.1371/journal.pntd.0010149)
Supplement: S2 Fig — Neonate C57BL/6 mice (6–8 days old) were infected by intradermal injection with 10 PFU, 102 PFU, and 106 PFU of CHIKV. (A) H&E staining of paraffin-embedded sections of contralateral feet was examined at 6 dpi. (B) H&E staining of paraffin-embedded sections of contralateral feet was also examined at 29 dpi. Original images are shown at low magnification (×50) and high magnification (×200). n = 5 per group. (DOCX) [file pntd.0010149.s003.docx]

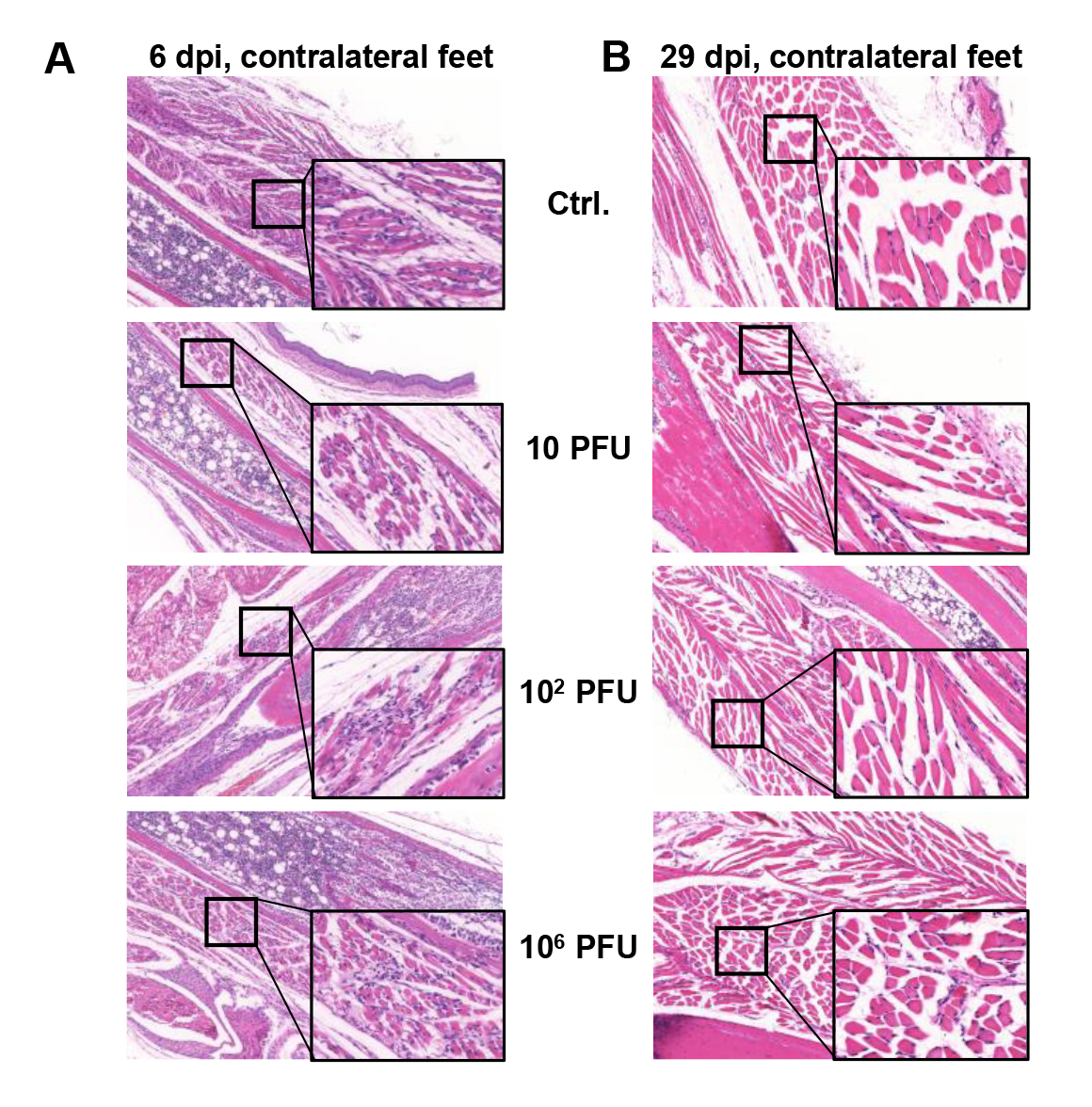


**S2 Fig. Pathological changes in the contralateral feet during the acute stage.** Neonate C57BL/6 mice (6-8 days old) were infected by intradermal injection with 10 PFU, 10^2^ PFU, and 10^6^ PFU of CHIKV. (A) H&E staining of paraffin-embedded sections of contralateral feet was examined at 6 dpi. (B) H&E staining of paraffin-embedded sections of contralateral feet was also examined at 29 dpi. Original images are shown at low magnification (×50) and high magnification (×200). n = 5 per group.
