# Supplementary material for: A high-dose inoculum size results in persistent viral infection and arthritis in mice infected with chikungunya virus
Source: PLoS Negl Trop Dis. 2022 Jan 31;16(1):e0010149. doi: 10.1371/journal.pntd.0010149 (PMC8803182; doi:10.1371/journal.pntd.0010149)
Supplement: S1 Table — (DOCX) [file pntd.0010149.s004.docx]

**S1 Table. Primers and probes of nsP1 and E1 for qRT-PCR detection**

| **Target** |  | **Sequence (5’-3’)** | **Position** |
| --- | --- | --- | --- |
| **nsP1 ^a [21]^** | Reverse transcription (positive strand) | GGCAGTATCGTGAATTCGATGCCGTGTCGGTAGTCTTGCACAT | nt 968 |
|  | Reverse transcription (negative strand) | GGCAGTATCGTGAATTCGATGCGCTAAAACCGTGCGACCGTGTGC | nt 704 |
|  | Sense | AAAGGGCAAGCTTAGCTTCAC | nt 806 |
|  | Antisense | GCCTGGGCTCATCGTTATTC | nt 893 |
|  | Probe | FAM/CGCTGTGACACAGTGGTTTCGTGTG/MGB | nt 831 |
| **E1 ^a [13]^** | Reverse transcription (positive strand) | GTGTGACGCCGGGTAATTGACTATA | nt 11095 |
|  | Reverse transcription (negative strand) | GTCAAATAGCAACAAACCCGGTAAG | nt 10689 |
|  | Sense | TCGACGCGCCATCTTTAA | nt 10785 |
|  | Antisense | AGTGCACTGCACACT | nt 10970 |
|  | Probe | FAM/ATCAGCCTGCACCCATTCCTCAGAC/ MGB | nt 10822 |

^a^ Numbering of the Sequence is according to KC488650.
