# Supplementary material for: A high-dose inoculum size results in persistent viral infection and arthritis in mice infected with chikungunya virus
Source: PLoS Negl Trop Dis. 2022 Jan 31;16(1):e0010149. doi: 10.1371/journal.pntd.0010149 (PMC8803182; doi:10.1371/journal.pntd.0010149)
Supplement: S2 Table — (DOCX) [file pntd.0010149.s005.docx]

**S2 Table. Primers for nested-RT-PCR amplification**

| **Target** |  | **Sequence (5’-3’)** | **Position** |
| --- | --- | --- | --- |
| **Fragment 1 ^a^** | Reverse transcription | CGGGAATGACACTCTTTCACCGTCTACC | nt 995 |
|  | Sense | AACCCATCATGGATTCTGTGTACGTGGA | nt 1 |
|  | Antisense | CGGGAATGACACTCTTTCACCGTCTACC | nt 995 |
|  | Nested-sense | ATGGATTCTGTGTACGTGGATAT | nt 9 |
|  | Nested-antisense | GCCTGGGCTCATCGTTATTC | nt 893 |
| **Fragment 2 ^a^** | Reverse transcription | ATTTTATATGGACAAGCGGGGCGAA | nt 2149 |
|  | Sense | AACCCATCATGGATTCTGTGTACGTGGA | nt 1 |
|  | Antisense | ATTTTATATGGACAAGCGGGGCGAA | nt 2149 |
|  | Nested-sense | AAAGGGCAAGCTTAGCTTCAC | nt 806 |
|  | Nested-antisense | ATTTTATATGGACAAGCGGGGCGAA | nt 2149 |
| **Fragment 3 ^a^** | Reverse transcription | TGGTATGTGGCCGCTTTAGCTGTTC | nt 9741 |
|  | Sense | AAGAAGAGTGGGTGACGCATAAGAA | nt 9381 |
|  | Antisense | TGGTATGTGGCCGCTTTAGCTGTTC | nt 9741 |
|  | Nested-sense | AAGAAGAGTGGGTGACGCATAAGAA | nt 9381 |
|  | Nested-antisense | CCACACCCACCATCGACAGGAGTAT | nt 9614 |

^a^ Numbering of the Sequence is according to KC488650.
